# Concomitant Effect of Quercetin- and Magnesium-Doped Calcium Silicate on the Osteogenic and Antibacterial Activity of Scaffolds for Bone Regeneration

**DOI:** 10.3390/antibiotics10101170

**Published:** 2021-09-27

**Authors:** Arul Murugan Preethi, Jayesh R. Bellare

**Affiliations:** 1 Department of Chemical Engineering, Indian Institute of Technology Bombay, Mumbai 400076, Maharashtra, India; 174020006@iitb.ac.in; 2 Wadhwani Research Center for Bioengineering (WRCB), Indian Institute of Technology Bombay, Mumbai 400076, Maharashtra, India

**Keywords:** quercetin, magnesium-doped calcium silicate, osteogenic activity, antibacterial activity, bone regeneration and nanofiber scaffold

## Abstract

Quercetin is a bioflavonoid which has a broad spectrum of biological activity. Due to its lower chemical stability, it is usually encapsulated, or a metal–quercetin complex is formed to enhance its biological activity at a lower concentration. Here, our novel approach was to form a quercetin complex to magnesium-doped calcium silicate (CMS) ceramics through a coprecipitation technique so as to take advantage of quercetin’s antibacterial activity within the antibacterial and osteogenic potential of the silicate. Due to quercetin’s inherent metal-chelating ability, (Ca+Mg)/Si increased with quercetin concentration. Quercetin in magnesium-doped calcium silicate ceramic showed concentration-dependent pro-oxidant and antioxidant activity in SaOS-2 with respect to quercetin concentration. By optimizing the relative concentration, we were able to achieve 3-fold higher proliferation and 1.6-fold higher total collagen at day 14, and a 1.7-fold higher alkaline phosphatase production at day 7 with respect to polycaprolactone/polyvinylpyrrolidone (PCL/PVP) scaffold. Quercetin is effective against Gram-positive bacteria such as *S. aureus*. Quercetin is coupled with CMS provided similar effect with lower quercetin concentration than quercetin alone. Quercetin reduced bacterial adhesion, proliferation and biofilm formation. Therefore, quercetin-coupled magnesium-doped calcium silicate not only enhanced osteogenic potential, but also reduced bacterial adhesion and proliferation.

## 1. Introduction

Designing resorbable scaffolds for bone tissue engineering is a multifactorial design problem. The current design aspect of scaffold design requires it to reduce/prevent microbial adhesion and growth and, if possible, kill the microbes, as well as aid in successful bone regeneration. Currently, many synthetic polymers such as polycaprolactone (PCL) do not possess inherent antibacterial property. Passive resistance against bacteria can be provided by making the scaffold hydrophilic. This can be achieved by including polyvinylpyrrolidone (PVP). Other routes to incorporate antibacterial properties into scaffold can be through the addition of nanoparticle and/or through antibiotics [1]. However, the excessive use of antibiotics leads to the development of antibiotic-resistant microbes. Therefore, one section of research focuses on identifying biomolecules that can offer properties such as those of antibiotics and also support tissue regeneration.

Quercetin (Q), a phenolic bioflavonoid which is predominantly found in vegetables such as onion, has antioxidant, anti-inflammatory, antibacterial and anti-viral properties [2]. Based on the cell type, quercetin can exhibit pro-oxidant or antioxidant effects [3]. Foundational requirements for successful bone regeneration are osteoblastogenesis and angiogenesis. When bone-marrow-derived mesenchymal stem cells (MSCs) were treated with 0.1 to 10 μM of quercetin, MSCs differentiated into osteoblast lineage by upregulating osteoblast-specific gene expression. Quercetin inhibits osteoclasts by reducing cell proliferation and resorption pits. Additionally, quercetin enhances angiogenesis by activating vascular endothelial growth factor signaling, upregulating angiogenin-1 [4]. Antibacterial mechanisms of flavonoids are different from those of conventional antibiotics. Quercetin exhibits antibacterial activity by decreasing bilayer thickness, opposing bacterial cell–cell signaling, inhibiting the biofilm of *E*. *coli* EAEC 042, inhibiting DNA gyrase, and preventing ATP hydrolysis [5]. The viability of *E*. *coli* and *S*. *aureus* when treated with 2 μg/mL of quercetin for 24 h was 40% and 60%. This reveals that quercetin did not offer complete bacterial growth inhibition. The efficacy was enhanced by cadmium complexing with N–N bidentate ligands [6].

Even though quercetin has a broad spectrum of biological activity, its chemical stability depends on pH, temperature, light and the oxidative environment. To enhance the chemical stability, thereby, bioavailability, suitable delivery systems have been designed. Their advantages and disadvantages are discussed elsewhere [7]. The sole purpose of many drug delivery system or vehicles is to release the bioflavonoid without affecting its chemical stability. However, many vehicles mostly do not possess inherent properties essential for osteoblast activity. Fabricating bioactive nanoparticles as delivery vehicles for bioflavonoids will enhance the overall biological performance.

Calcium-silicate-based ceramic nanoparticles demonstrate excellent bioactivity. Their degradation rate can be fine-tuned by doping elements such as magnesium. This inherently upregulates osteoblast activity. There are various synthesis techniques for magnesium-doped calcium silicate (CMS) ceramics [8]. Commonly used techniques such as coprecipitation are flexible to accommodate bioflavonoids without compromising their chemical stability. The objective of this article is to investigate the effect of quercetin–CMS systems on their ability to enhance osteoblast activity using human osteosarcoma (SaOS-2) cell lines and on their ability to resist bacterial adhesion and proliferation.

## 2. Results

This section discusses nanoparticle and scaffold characterization under two different subsections.

### 2.1. Nanoparticle Characterization

**Figure 1:**
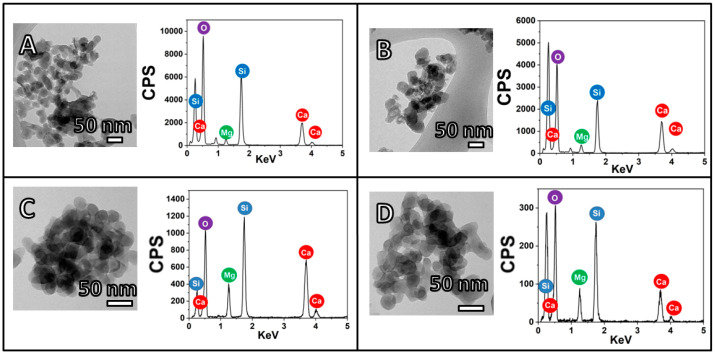
TEM and EDX of (**A**) CMS, (**B**) CMSQ5, (**C**) CMSQ10 and (**D**) CMSQ20 nanoparticles. The shapes of all nanoparticles are nano-plate. EDX confirmed the presence of calcium, magnesium and silicate ions.

**Figure 2:**
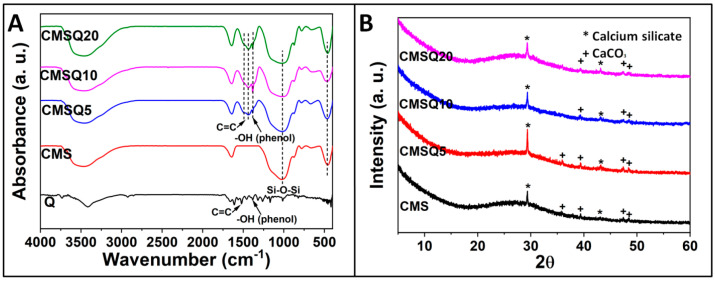
(**A**) FTIR and (**B**) XRD of CMS, CMSQ5, CMSQ10, and CMSQ20 nanoparticles. FTIR confirmed the presence of quercetin in its structure. XRD showed the presence of calcium silicate and CaCO_3_ peaks.

Magnesium-doped calcium silicate (CMS) ceramics prepared through coprecipitation technique rendered them as nano-plate-like structures, as shown in Figure 1. The structure did not change upon quercetin addition. Incorporation of quercetin increased the Mg/Ca ratio in the nanoparticle, and (Ca+Mg)/Si was also found to be higher for the CMSQ10 nanoparticle (Table 1). FTIR spectra (Figure 2A) of the CMS nanoparticle exhibited Si–O–Si antisymmetric stretching at ~1080 to 1095 cm^−1^ and symmetric stretching at ~465 cm^−1^. FTIR spectra of quercetin show C=C stretching in the aromatic ring at 1560 cm^−1^ and –OH (phenolic group) at 1379 cm^−1^. These quercetin functional groups were detected in CMSQ5, CMSQ10 and CMSQ20 nanoparticles, confirming the presence of quercetin in its structure [9]. XRD patterns of all the nanoparticles (Figure 2B) showed that nanoparticles are amorphous in nature, and peaks at around 29° showed the characteristic peak of calcium silicate [10]. Peaks of CaCO_3_ appeared because the nanoparticles were prepared in an ambient environment.

### 2.2. Scaffold Characterization

**Figure 3:**
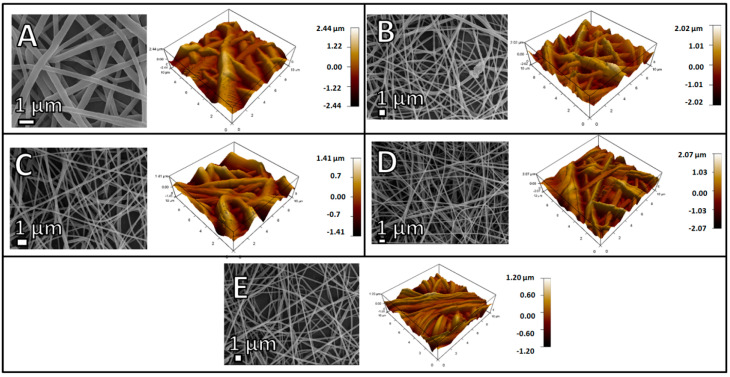
SEM and AFM images of (**A**) P (**B**) PCMS, (**C**) PCMSQ5, (**D**) PCMSQ10 and (**E**) PCMSQ20 scaffolds. Incorporation of a biomolecule loaded and unloaded with CMS nanoparticles significantly changed the nanofiber diameter and scaffold roughness.

**Figure 4:**
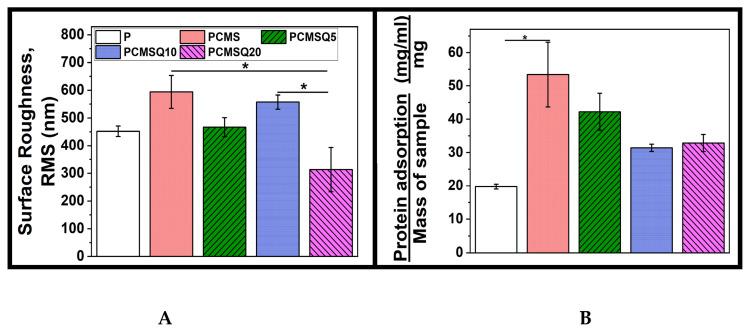
(**A**) Surface roughness and (**B**) protein adsorption of the scaffold. Nanofiber arrangement altered scaffold roughness. The presence of quercetin affected BSA adsorption. * stand for *p* < 0.05.

**Figure 5:**
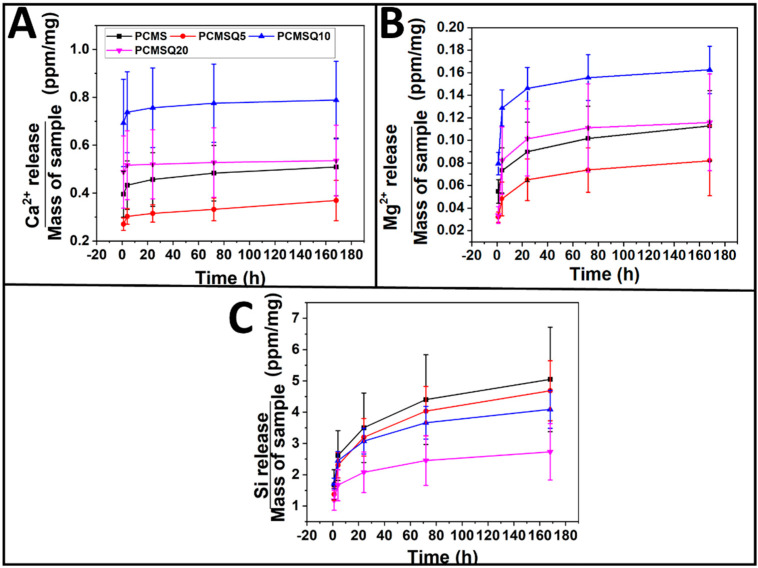
Ion release from the scaffold upon immersion in DI water. (**A**) Calcium ion release, (**B**) magnesium ion release, and (**C**) silicon ion release from the scaffold. Release of calcium and magnesium ion was similar across all the scaffolds, but silicon ion release decreased with increasing quercetin into the nanoparticle.

The scaffold notation PCMSQx (where x = 0, 5, 10 and 20 mg of quercetin per 100 mL of total nanoparticle precursor solution) indicates the electrospun scaffold using PCL/PVP as a polymer that had a quercetin-coupled CMS nanoparticle. Electrospinning parameters for the scaffold are tabulated in Table 2. Addition of the nanoparticle onto the polymer solution altered solution viscosity and conductivity, which, in turn, required a higher flow rate which affected the nanofiber diameter and nanofiber orientation, followed by scaffold surface roughness (Figure 3). PCMSQ20 had the smallest nanofiber diameter that affected the roughness of the scaffold (Figure 4B). A protein adsorption study was performed to evaluate the scaffold’s ability to attract blood protein upon insertion, thereby starting the cascade of the bone regeneration process. From Figure 4B, it can be observed that with the increasing concentration of quercetin in CMS, protein adsorption reduced. This is due to the hydrophobic nature of quercetin and its strong affinity to BSA protein [11]. With increasing concentrations of quercetin in CMS, more quercetin will be released, which will then bind to a hydrophobic BSA protein, preventing it from further adsorption on to the scaffold surface (Figure 4). From the ion release study (Figure 5), it was revealed that the amorphous nature of nanoparticles supported ion release. Additionally, quercetin interacted with all the ions effectively. With the increase in quercetin concentration, the sustained release of silicate ion was observed, because quercetin bonded with silicon.

**Figure 6:**
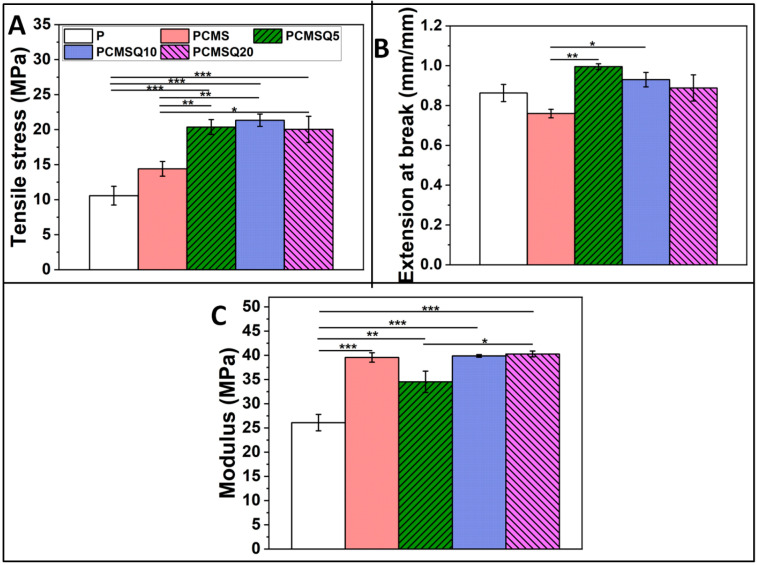
(**A**) Tensile stress, (**B**) extension at break, and (**C**) modulus of P, PCMS, PCMSQ5, PCMSQ10 and PCMSQ20 scaffolds. Presence of the nanoparticle improved strength of the scaffold, followed by the modulus. *, ** and *** stand for *p* < 0.05, 0.01 and 0.001.

To study the mechanical property of the scaffold, the tensile stress, extension at break and modulus was analyzed. It was observed that the tensile stress and modulus of the scaffold increased with the addition of CMS. The presence of CMS-based nanoparticle into the scaffold significantly enhanced the load-bearing ability of the scaffold by restricting the polymer elongation (Figure 6).

**Figure 7:**
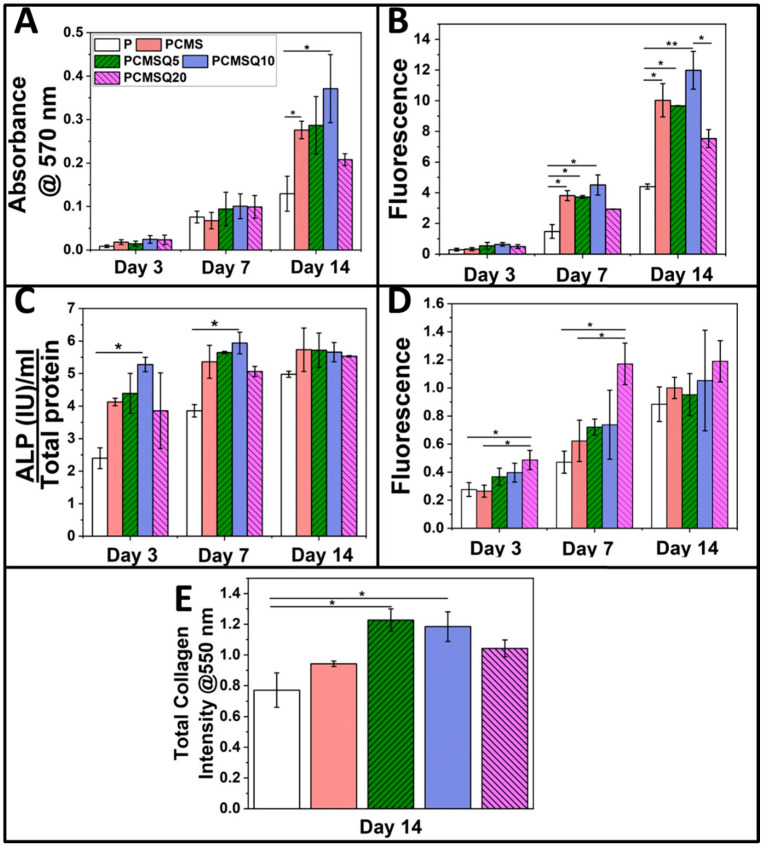
Osteogenic potential assessment (**A**–**E**). (**A**) MTT assay, (**B**) DNA quantification, (**C**) ALP (alkaline phosphatase), (**D**) ROS production, and (**E**) total collagen quantification of SaOS-2. Quercetin coupled with CMS increased the osteogenic activity by enhancing osteogenic markers such as ALP and collagen production. The maximum acceptable quercetin in the system that exhibited osteogenic properties is PCMSQ10. * and ** stand for *p* < 0.05 and 0.01.

**Figure 8:**
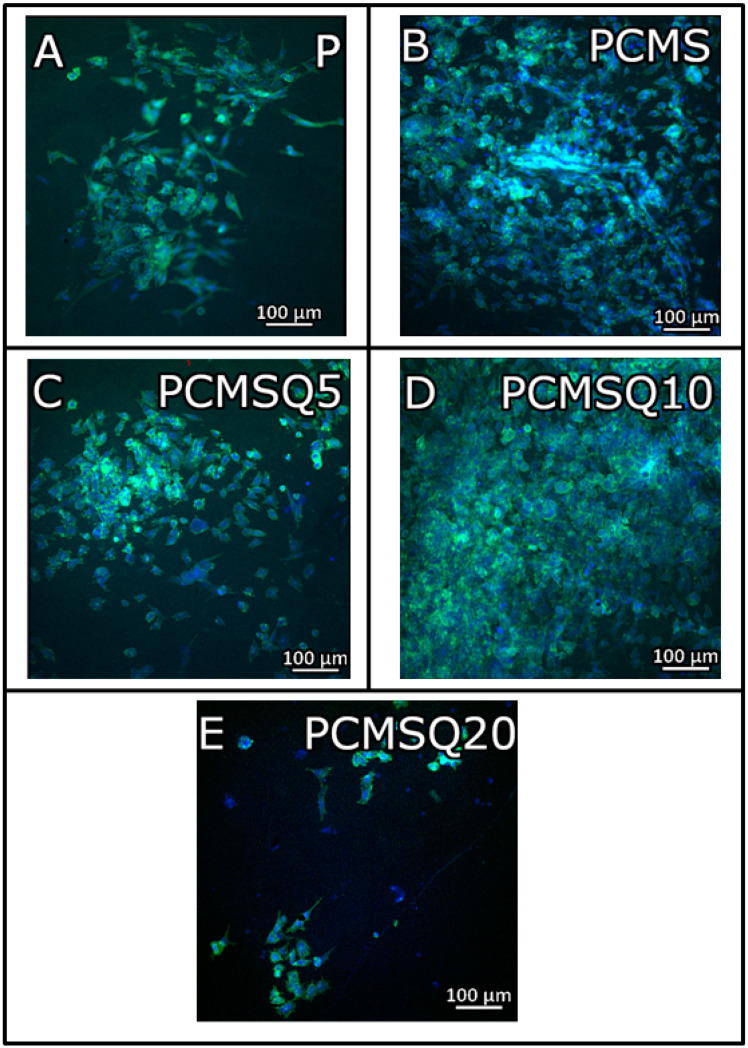
Confocal image of (**A**) P, (**B**) PCMS, (**C**) PCMSQ5, (**D**) PCMSQ10 and (**E**) PCMSQ20 scaffolds at day 14 confirms that PCMSQ10 had enhanced osteogenic propertied. The confocal images corroborated the MTT and DNA quantification analyses.

The osteogenic potential of the scaffold was tested with the MTT assay (Figure 7A), DNA quantification (Figure 7B), ALP quantification (Figure 7C), ROS generation (Figure 7D) and total collagen synthesis (Figure 7E) using the SaOS-2 cell line. An in vitro study of quercetin and the CMS system in the PCL/PVP scaffold revealed that the presence of quercetin did not affect the osteogenic potential of CMS. The maximum osteogenic potential in the form of higher proliferation, ALP and collagen synthesis was observed for the PCMSQ10 scaffold. With further increases in quercetin concentration, intercellular reactive oxygen species (ROS) production was increased, which affected the osteogenic potential of the scaffold. The in vitro data were corroborated with confocal images, as shown in Figure 8.

**Figure 9:**
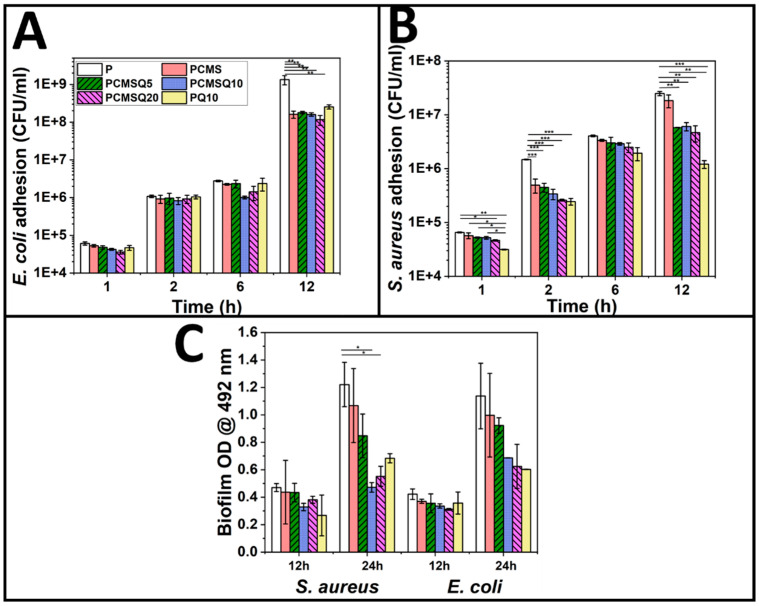
Antibacterial potential assessment of the scaffold. Kinetic study of adhered bacteria (**A**) *E. coli* and (**B**) *S. aureus* on the scaffold. (**C**) Biofilm quantification on the scaffold at 12 h and 24 h. Quercetin is effective against *S. aureus*. Coupling magnesium-doped calcium silicate and quercetin reduced *S. aureus* adhesion and biofilm formation at lower quercetin concentrations. *, ** and *** stand for *p* < 0.05, 0.01 and 0.001.

The antibacterial activity of the scaffold was analyzed by using *E. coli* and *S. aureus*. The scaffold was placed in an environment that supported bacterial proliferation (Figure 9). It was observed that the presence of CMS alone in the scaffold reduced *E. coli* adhesion by 1 log factor, whereas for quercetin alone, it required 10% loading on PCL/PVP (PQ10) to provide a similar effect. When quercetin was coupled with CMS, with reduced quercetin loading, bacterial adhesion was reduced by 1 log factor, which was mainly due to CMS but did not reduce the biofilm formation significantly.

**Figure 10:**
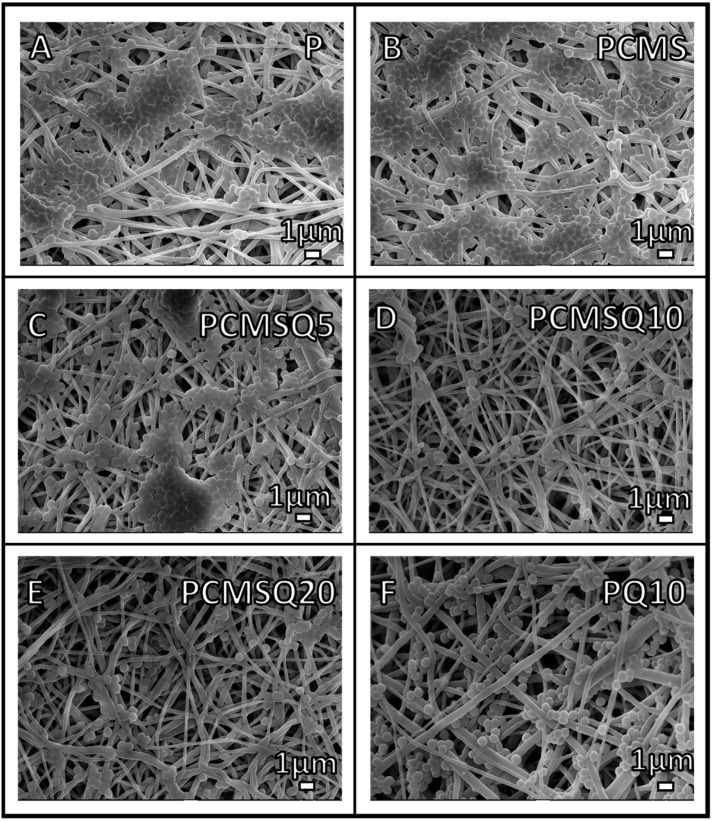
SEM images of *S. aureus* on (**A**) P, (**B**) PCMS, (**C**) PCMSQ5, (**D**) PCMSQ10, (**E**) PCMSQ20 and (**F**) PQ10 scaffolds at 24 h. The PCMSQ10 scaffold had the fewest adhered bacteria compared to the other scaffold. The SEM images correlated well with the OD of the biofilm.

However, quercetin is effective against Gram-positive bacteria and significantly reduced the proliferation and biofilm formation in *S. aureus* (Figure 9B). The coupling effect of CMS and quercetin in the PCMSQ10 scaffold reduced biofilm formation by 2.4-fold compared to scaffold P at 24 h. The SEM of the scaffold with *S. aureus* (Figure 10) corroborated the result, as shown in Figure 9E.

## 3. Discussion

Scaffold design for bone tissue regeneration along with local antibiotic therapy is an important research area that aims to avoid post-operative infection leading to osteomyelitis. Conventional antibiotic-loaded scaffold eliminates microbes at the cost of developing antibiotic-resistant bacteria, and it also requires additional compounds that impart osteogenic potential to the scaffold [1]. The research gap can be bridged by finding a suitable scaffold system that reduces bacterial adhesion along with improving the osteogenic activity.

Synthetic polymer scaffolds made out of PCL in general do not possess antibacterial properties [1]. They are either blended or modified with a polymer that can provide at least passive resistance to bacterial adhesion. Polymers such as polyamides, polyethylene glycol, etc., provide antibiofouling effects through passive resistance [1]. PVP is also one such polymer that exhibits antibiofouling effects, whose incorporation increases hydrophilicity to the scaffold that not only improves osteoblast adhesion, but also decreases bacterial adhesion. Their solubility in water provides a suitable platform for the enhanced availability of molecules for drug-delivery-based scaffolds [12].

Flavonoids such as quercetin are known to exhibit a spectrum of biological activity. They are synthesized by plants in response to microbial attack. Quercetin has found its foothold in nerve [13], skin tissue engineering applications [14] and also in cancer treatment [15]. Recently, studies on quercetin-based scaffolds in bone tissue engineering are gaining attention among researchers because of quercetin’s multifaceted properties. The dose-dependent activity of quercetin is cell-line-specific. Quercetin at a lower concentration supports cell proliferation in nerve [13], skin [14] and bone scaffolds, but at higher concentrations, quercetin acts as an anticancer agent [15]. Therefore, it is important to administer appropriate dosages to gain the potential of quercetin-based scaffolds.

Bone-marrow-derived MSCs cultured on quercetin inlaid silk hydroxyapatite scaffolds revealed that the lowest quercetin concentration, i.e., 0.03 wt.%, had the highest ALP production and COL-I and Runx2 gene expression in vitro. In vivo studies on rat calvaria also confirmed that, at 0.03 wt.%, the bone mineral density, bone volume and fraction were found to be higher [16]. When MC3T3-E1 cells were cultured on a 3D-printed polydopamine-poly (l-lactide) scaffold, it was revealed that osteogenic activity such as cell proliferation, ALP and mineralization was found to be higher for coating concentrations up to 200 μM. However, the osteogenic activity was lower for coating concentrations of 400 μM [17]. Similarly, a poly (l-lactide) chitosan scaffold coated with polydopamine and 200 μM of quercetin exhibited higher cell proliferation, ALP and mineralization by MC3T3-E1 cells [18]. The presence of –OH groups in quercetin helps them effectively chelate with metal ions. A zinc (quercetin)(phenanthroline) complex in PCL/gelatin scaffold enhanced angiogenic and osteogenic activity [19]. Similar effects were found in a copper (quercetin)(phenanthroline) complex and copper (quercetin)(neocuproine) complex [20]. From the above metal–quercetin complex study, it can be understood that MG-63 cells treated with quercetin concentrations above 80 μM show cytotoxic effect. However, the cytotoxic limit was reduced to 60 μM. 

Quercetin exhibits better antibacterial activity towards Gram-positive bacteria. Due to their poor water solubility and low chemical stability, they are chemically modified to improve their antibacterial performance [21,22]. To enhance the quercetin’s antibacterial activity, it needs to be complexed with metal ions. Quercetin complexed with Mn^2+^, Hg^2+^, Co^2+^ and Cd^2+^ showed antibacterial activity against *S. aureus*, *Bacillus cereus*, *P. aeruginosa*, *E. coli*, and *Klebsiella pneumonia* than quercetin at similar concentrations [5]. 

Thus far, to the best of our knowledge, no studies have focused on the dual properties (i.e., osteogenic and antibacterial activity) of quercetin in bone tissue engineering. Quercetin expresses both pro-oxidant and antioxidant effects [3]. Therefore, optimizing quercetin concentration that provides the scaffold with dual property has been the aim of this article.

In our study, the osteogenic potential of quercetin showed dose-dependent behavior. The coupling of CMS and quercetin increased the scaffold’s osteogenic activity up to the PCMSQ10 scaffold, i.e., the proliferation and total collagen production were 3-fold and 1.6-fold higher than PCL/PVP at day 14, respectively. With any further increase in quercetin concentration in the scaffold, the scaffold started showing signs of reduced cell viability and osteogenic potential by increasing the ROS production. Similar effects were found in scaffolds used for neural repair [13]. We were able to achieve higher osteogenic potential at the lowest quercetin concentration compared to the concentration reported in the literature. This was due to the presence of calcium, magnesium and silicon ions along with quercetin in the scaffold, which provided favorable outcomes in osteoblast activity.

The antibacterial potential of the scaffold was evaluated using *E. coli* and *S. aureus*. CMS quercetin system showed better antibacterial potential towards *S. aureus* from the start of the experiment than *E. coli*. This reveals that Gram-positive bacteria are susceptible to quercetin even at the lowest concentration than Gram-negative bacteria because quercetin affects the cell membrane permeability and integrity of *S. aureus* [23]. At lower quercetin concentrations, antibacterial activity against *E. coli* comes from CMS alone. This may be because the concentration of quercetin inside the scaffold is too low to provide any antibacterial activity against *E. coli*. Our data corroborate the literature which conveys that the minimum inhibition concentration of quercetin towards *S. aureus* is much lower than that of *E. coli* [20]. The mode of antibacterial activity in *S. aureus* can be due to outbursts of reactive oxygen species and decreases in the proton-motive force in *S. aureus* that affects the membrane permeability [18]. 

Therefore, our study addressed the important aspect of bridging the anti-microbial research gap by formulating a biomolecule-based scaffold and demonstrating that, it has both osteogenic and antibacterial activity.

## 4. Experimental 

### 4.1. Materials

Polycaprolactone (PCL, M.W. 80,000 Da), sodium silicate solution (Extra pure), 3-(4,5-dimethylthiazol-2-yl)-2,5-diphenyltetrazolium bromide (MTT) and Direct Red 80 was purchased from Sigma-Aldrich, India. Polyvinylpyrrolidone (PVP, M.W. 40,000 Da), magnesium nitrate hexahydrate (98% purity), McCoy’s 5A media with ι-glutamine, fetal bovine serum (FBS, gamma-irradiated, sterile-filtered, South American) and Antibiotic Antimycotic Solution 100X Liquid (w/10,000 U penicillin, 10 mg streptomycin and 25 µg amphoteric B per ml in 0.9% normal saline) was purchased from Himedia, India. Calcium nitrate tetrahydrate (99% purity) was purchased from S D fine-Chem limited, India. GlutaMAX^TM^-1(100X) was purchased from Thermofisher Scientific, India. Picric acid extrapure AR, 99.8% was purchased from SRL Pvt. Ltd., Mumbai, India. All chemicals were used as purchased.

### 4.2. Sample Preparation

#### 4.2.1. One-Pot Synthesis of Quercetin in Calcium Magnesium Silicate

Solution A containing 0.09 M calcium nitrate tetrahydrate and 0.01 M magnesium nitrate hexahydrate was adjusted to pH 11, before it was mixed with solution B containing 0.1 M sodium silicate. The entire solution was mixed for 30 min. The precipitate was washed and dried overnight. For the quercetin loading, 5, 10, and 20 mg of quercetin per 100 mL of total solution was added to solution B and was then mixed with solution A. Nanoparticles with 0, 5, 10, and 20 g of quercetin/mL of total solution were named as CMS, CMSQ5, CMSQ10 and CMSQ20, respectively. Elemental compositions of nanoparticles are given in Table 1.

#### 4.2.2. Electrospinning Solution Preparation and Nanofiber Fabrication

The solution for electrospinning consisted of a solvent comprising 2.5 mL dichloromethane and 1 mL methanol, and the polymer comprised 0.25 g of PCL and 0.05 g of PVP. The solution was vigorously mixed for 1 h. The nanofiber mat was prepared using a 2ml syringe and 23 G blunt needle (BD Discardit^TM^ II syringe, India) in an electrospinning machine (ESPIN NANO, India) and is denoted as “P”. To prepare nanoparticle-incorporated nanofiber mats, 5 wt.% of nanoparticles with respect to PCL was added to the above solution and the scaffolds were named as PCMS, PCMS5Q, PCMS10Q and PCMS20Q, respectively. All the scaffolds were vacuum-dried at room temperature in a desiccator; the parameters for electrospinning are given in Table 2.

### 4.3. Characterization

#### 4.3.1. Scanning Electron Microscopy (SEM)

Parameters for electrospinning were optimized with the help of field emission gun scanning electron microscopy (JEOL JSM-7600F FEGSEM). All the samples were sputter-coated with a 10 nm thickness of platinum before analysis.

#### 4.3.2. Transmission Electron Microscopy (TEM)

Morphology and elemental analyses of the nanoparticles were performed with JEOL, JEM 2100F and energy-dispersive X-ray spectroscopy in combination with TEM. To prepare the sample for analysis, a few milligrams of nanoparticles were added to isopropanol and sonicated for 30 min. Then, a few droplets of the above solution were drop-casted on top of the carbon-coated copper grid and dried.

#### 4.3.3. Atomic Force Microscopy (AFM)

AFM (MFP3D Origin, Asylum/Oxford Instruments) was used to analyze the surface roughness of the scaffold. The scan range was 10 × 10 µm^2^ with a frequency of 0.5/Hz.

#### 4.3.4. Fourier-Transfer Infrared Spectroscopy (FTIR)

The nanoparticle functional groups were analyzed using a 3000 Hyperion Microscope with Vertex 80 FTIR system (Bruker, Germany) in the range of 4000–400 cm^−1^.

#### 4.3.5. X-ray Diffraction (XRD)

The phase and crystallinity of nanoparticles were analyzed using a Rigaku Smartlab X-ray diffractometer with a 3 kW X-ray generator Cu tube. The analysis was performed between 2θ of 5° to 60° at room temperature with a scan rate of 0.05°/s.

#### 4.3.6. Ion Release Study

The scaffold was pre-weighed and immersed in 5 mL deionized water at 37 °C (DI water). This scaffold was re-immersed into DI water for every time point. The liquid samples were then analyzed using inductively coupled plasma–atomic emission spectroscopy, ICP-AES (ARCOS, Simultaneous ICP Spectrometer, SPECTRO Analytical Instruments GmbH, Kleve, Germany). 

#### 4.3.7. Quantification of Protein Adsorption on the Scaffold

This analysis was performed to understand the effect of quercetin on the scaffold’s ability to adsorb bovine serum albumin (BSA). The scaffolds of size of 1 × 1 cm^2^ were immersed in a 1ml solution containing 5 g/dL of BSA and were incubated for 2 h at 37 °C. Next, the scaffold after incubation was immersed in 1% sodium dodecyl sulfate solution (SDS) for 2 h to strip adsorbed BSA away. Later, the BSA protein in SDS was quantified using Micro BCA Protein Assay Kit 23235 (ThermoFisher Scientific, Mumbai, India), as per the manufacturer’s protocol.

#### 4.3.8. Tensile Test

Uniaxial tensile testing of the scaffolds was performed using an Instron 2519 series using a 5 kN load cell at room temperature with a strain rate of 5 mm/min. Analysis was performed according to ASTM D882 standards.

### 4.4. In Vitro Assessment of Scaffold Using SaOS-2

The human osteosarcoma SaOS-2 cell line was purchased from NCCS, Pune, India, and was cultured using McCoy’s 5A media containing ι-glutamine containing 1% GlutaMAX^TM^-1(100X), 1% antibiotic and antimycotic solution. The cells were maintained in a humidified incubator kept at 37 °C with 5% carbon dioxide (CO_2_). For the assays, the scaffolds were placed in non-tissue-culture-treated 24-well plate (Eppendorf USA), and sterilized using 70% ethanol and UV for 1 h each. The scaffolds were preconditioned using cell culture media for 1 h each. Cells were seeded at a density of 4 × 10^4^ cells per cm^2^ and incubated for 4 h at 37 °C with 5% CO_2_. Afterwards, 0.4 mL media were added to all the wells, and replenished every 3 days.

Cell viability on the scaffold was evaluated using (3-(4,5-Dimethylthiazol-2-yl)-2,5-Diphenyltetrazolium Bromide) solution (MTT, 1 mg/mL in PBS). After culturing cells until a predetermined time point, the scaffolds were washed with PBS and then incubated in 200 μL of MTT solution for 4 h, followed by adding 800 μL of DMSO to dissolve the formazan crystals. The optical density at 470 nm was measured using MultiskanSkyHigh Microplate Spectrophotometer (Thermo-Fischer Scientific, Mumbai, India).

Cell proliferation on the scaffold was assessed using a Quant-iT Pico Green DNA assay kit. After culturing cells to a predetermined time point, the scaffolds were washed with PBS and then freeze–thawed twice in 500 μL of autoclaved deionized water. The solution was then centrifuged at 10,621× *g* for 10 min; then, 100 μL of cell lysate supernatant was mixed with 100 μL of Picogreen working solution and incubated for 5 min, as per the manufacturer’s protocol. Fluorescence intensity was measured using a Varioskan LUX Multimode Microplate Reader at 490/538 nm, respectively.

To measure the earlier marker for osteogenic maturation (alkaline phosphatase), a Sensolyte^®^ pNPP Alkaline Phosphatase Assay colorimetric kit was used. The assay was performed as per the manufacturer’s protocol.

SaOS-2 cells majorly produce collagen type-I, which is also a marker for osteoblast differentiation. To economically quantify total collagen, picosirius red dye is used. In this study, 1 × 10^5^ cells were seeded on top of the 1.5 × 1.5 cm^2^ scaffold in 12-well plate for 14 days. After culturing cells for 14 days, cells were lysed using 0.2% Triton X-100 prepared in autoclaved deionized water, followed by freeze–thawing and centrifugation (4 °C at 2500 rpm for 10 min). To 100 μL of cell lysate supernatant, 900 μL of Sirius red solution (0.1% direct red dye 80 in saturated picric acid) was mixed for 30 min followed by centrifugation (10 min at 14,000 rpm). The supernatant was discarded, and 500 µL of 0.5 N NaOH was added to the pellet and then vortexed for 10 min followed by measuring the optical density at 550 nm.

Cells on the scaffold were imaged using spinning disc confocal microscopy (Yokogawa Electric Corporation, CSU-X1). Cells in the scaffolds were permeabilized using 0.2% Triton-X 100. Actin filaments were stained using 2 units/mL of FITC-phallodin, and the nucleus was stained using 10 µg/mL of DAPI. Later, images were processed through Zen software (Zeiss).

### 4.5. In Vitro Adhesion Assessment of E. coli and S. aureus on Scaffold

Microorganisms (*E. coli* K12 or *S. aureus* MTCC 96) at 0.1 optical density (at 600 nm) in LB broth were added to the scaffold and incubated at 37 °C for 1 h, 2 h, 6 h and 12 h. Later, the scaffold was dipped in 1 mL PBS, sonicated for 10 min, and vortexed for 1 min to detach the adhered microorganism from scaffold and was diluted with PBS. After diluting several times, the bacteria were grown on agar plates and the colony-forming units per ml were counted. One set of adhered bacteria on the scaffold was dehydrated by serially increasing the concentration of ethanol and dried at 37 °C for 12 h and imaged under SEM.

### 4.6. Biofilm Formation on the Scaffold

Biofilm formation on the scaffold was quantified by the tissue culture plate method [24]. Scaffolds were placed in LB broth for 12 h and 24 h. Later, the microorganisms on the scaffolds were fixed using glutaraldehyde (2.5%) at 4 °C for 30 min and dried at 60 °C for 1 h. The biofilms were stained using crystal violet (500 µL, 0.1%) solution at room temperature for 20 min, followed by rinsing with PBS and drying at 37 °C. Stained biofilms on the scaffold were dissolved in 500 µL of 2% acetic acid for 15 min under gentle agitation. The OD at 492 nm was measured using a microplate spectrophotometer.

### 4.7. Statistical Analysis

Data presented in this article are the result of at least triplicates of every experiment, expressed as the mean ± standard deviation, and analyzed using one-way analysis of variance (ANOVA). Significant differences were calculated using Tukey’s test and are represented as *, ** and *** for *p* < 0.05, 0.01 and 0.001, respectively.

## 5. Conclusions

In our study, quercetin-coupled CMS nanoparticles were prepared through the coprecipitation technique. Quercetin chelated well with ions by increasing the (Ca+Mg)/Si ratio. Nanoparticles were incorporated in the PCL/PVP matrix and electrospun to produce a nanofibrous scaffold. Incorporation of this nanoparticle improved the tensile stress and modulus of the scaffold. The effect of quercetin-coupled CMS was optimized to provide improved osteogenic activity by enhancing the proliferation of SaOS-2, ALP and collagen synthesis, and inhibiting the proliferation of both *E. coli* and *S. aureus* on the scaffold with reduced quercetin loading, making it an attractive material for studies in bone tissue engineering.

## Figures and Tables

**Table 1:** Elemental composition of nanoparticles as per TEM.

Sl. No.	Sample Code	Elements	(Ca+Mg)/Si	Mg/Ca
1	CMS	Ca, Mg, Si	0.44 ± 0.09	0.28 ± 0.07
2	CMSQ5	Ca, Mg, Si	0.65 ± 0.13	0.28 ± 0.14
3	CMSQ10	Ca, Mg, Si	1.06 ± 0.06	0.48 ± 0.05
4	CMSQ20	Ca, Mg, Si	0.61 ± 0.04	0.69 ± 0.28

**Table 2:** Optimized electrospinning parameters of the scaffolds and their fiber diameter.

Sl. No.	Sample Code	Flowrate (mL/h)	Voltage (KV)	Distance (cm)	Fiber Diameter (nm)
1	P	0.5	25	23	0.56 ± 0.14
2	PCMS	1.2	19	21	0.31 ± 0.07
3	PCMS5Q	1.2	17	17	0.35 ± 0.11
4	PCMS10Q	1.2	19	17	0.24 ± 0.06
5	PCMS20Q	1.2	21	21	0.29 ± 0.06

## Data Availability

The data can be available on request to the corresponding author.
